# Developing a machine learning-based prognosis and immunotherapeutic response signature in colorectal cancer: insights from ferroptosis, fatty acid dynamics, and the tumor microenvironment

**DOI:** 10.3389/fimmu.2024.1416443

**Published:** 2024-07-15

**Authors:** Junchang Zhu, Jinyuan Zhang, Yunwei Lou, Yijie Zheng, Xuzhi Zheng, Wei Cen, Lechi Ye, Qiongying Zhang

**Affiliations:** ^1^ Department of Colorectal and Anal Surgery, The First Affiliated Hospital of Wenzhou Medical University, Wenzhou, China; ^2^ Department of Gastroenterology, The First Affiliated Hospital of Wenzhou Medical University, Wenzhou, China; ^3^ Department of Pathology, The First Affiliated Hospital of Wenzhou Medical University, Wenzhou, China

**Keywords:** ferroptosis, fatty acid metabolism, TME, colorectal cancer, immunotherapy, machine learning

## Abstract

**Instruction:**

Colorectal cancer (CRC) poses a challenge to public health and is characterized by a high incidence rate. This study explored the relationship between ferroptosis and fatty acid metabolism in the tumor microenvironment (TME) of patients with CRC to identify how these interactions impact the prognosis and effectiveness of immunotherapy, focusing on patient outcomes and the potential for predicting treatment response.

**Methods:**

Using datasets from multiple cohorts, including The Cancer Genome Atlas (TCGA) and Gene Expression Omnibus (GEO), we conducted an in-depth multi-omics study to uncover the relationship between ferroptosis regulators and fatty acid metabolism in CRC. Through unsupervised clustering, we discovered unique patterns that link ferroptosis and fatty acid metabolism, and further investigated them in the context of immune cell infiltration and pathway analysis. We developed the FeFAMscore, a prognostic model created using a combination of machine learning algorithms, and assessed its predictive power for patient outcomes and responsiveness to treatment. The FeFAMscore signature expression level was confirmed using RT-PCR, and ACAA2 progression in cancer was further verified.

**Results:**

This study revealed significant correlations between ferroptosis regulators and fatty acid metabolism-related genes with respect to tumor progression. Three distinct patient clusters with varied prognoses and immune cell infiltration were identified. The FeFAMscore demonstrated superior prognostic accuracy over existing models, with a C-index of 0.689 in the training cohort and values ranging from 0.648 to 0.720 in four independent validation cohorts. It also responses to immunotherapy and chemotherapy, indicating a sensitive response of special therapies (e.g., anti-PD-1, anti-CTLA4, osimertinib) in high FeFAMscore patients.

**Conclusion:**

Ferroptosis regulators and fatty acid metabolism-related genes not only enhance immune activation, but also contribute to immune escape. Thus, the FeFAMscore, a novel prognostic tool, is promising for predicting both the prognosis and efficacy of immunotherapeutic strategies in patients with CRC.

## 1 Introduction

Colorectal cancer (CRC) is one of the most prevalent malignant tumors of the digestive system. According to the American Cancer Society, approximately 81,860 patients with CRC were diagnosed and 28,470 deaths occurred in the United States of America in 2023, causing serious problems for patients and public health (1). Although endoscopic screening has reduced the mortality and morbidity rates of CRC in recent years, and recurrence and metastasis remain major challenges (2). Currently, primary treatments for CRC include surgery, chemotherapy, and radiotherapy. Nevertheless, advances in immunotherapies, including anti-PD-1, anti-PD-L1, and anti-CTLA4 treatments, have presented a new and promising therapeutic paradigm for CRC with significant potential efficacy (3). For instance, the successful anti-PD-1 application in patients with CRC and mismatch repair deficiency (dMMR) or high microsatellite instability (MSI-H) significantly causes progression-free survival in CRC (4, 5). A new combination of radiotherapy and immunotherapy promotes robust antitumor immune priming (6, 7). However, these methodologies face constraints arising from spatiotemporal heterogeneity, moderate precision, or limited representation of population subsets (8–10). Consequently, in the context of personalized treatment paradigms, the identification of robust biomarkers is essential for optimizing prognosis and enhancing the efficacy of drug therapies for CRC.

Ferroptosis, driven by biochemical and genetic components, is a programmed cell death pathway reliant on iron and activated by lipid peroxide buildup on cellular membranes. Its involvement extends to tumor advancement and therapeutic responses across various malignancies and is often intertwined with reactive oxygen species (ROS) that participate in cancer-related pathways (10). Fatty acid metabolism is a crucial cellular process that transforms nutrients into metabolic intermediates used for membrane synthesis, energy reservation, and signaling molecule production. This process has garnered significant attention as a potential target for cancer therapy, particularly because it is associated with regulatory and CD8+ T cells (11–13). Glutathione peroxidase 4 (GPx4) and prolyl hydroxylase-3 (PHD3) represent significant regulators of ferroptosis and fatty acid metabolism, respectively, emphasizing the potential role of both in immunotherapy (14, 15). Recent studies indicated a significant association between fatty acid metabolism and ferroptosis. Microsomal triglyceride transfer protein (MTTP)expression increases in the body during fatty acid metabolism, which inhibits ferroptosis and decreases the density of chemotherapy (16). Similar results were observed for phospholipids containing a single polyunsaturated fatty acyl tail (PL-PUFA1s), which are also strongly correlated with ferroptosis (17). Additionally, cytochrome P450 1B1 (CYP1B1) and cyclin-dependent kinase 1 (CDK1) degrade acyl-CoA synthetase long-chain family member 4 (ACSL4), who plays an essential role in fatty acid metabolism and inhibits ferroptosis, thereby inducing resistance to anti-PD-1 and oxaliplatin, respectively (18, 19). Thus, the fatty acid metabolism-related genes appear to regulate ferroptosis and function as intermediates. The relationship between ferroptosis regulators and fatty acid metabolism-related genes, which may significantly influence prognosis and drug resistance in colorectal cancer, has been less explored. The tumor microenvironment (TME), which consists of tumor cells, stromal cells, and immune cells, plays an irreplaceable role in the metastasis and tumor progression and also affects the efficacy of immune checkpoint blockade (ICB) treatment (20, 21). Considering the special relationship between ferroptosis and fatty acid metabolism regulators, a unique TME may induce novel metabolic pathways in CRC. Thus, the interactions between ferroptosis and fatty acid metabolism regulatory molecules should be explored in multicenter cohorts from a multi-omics perspective, including the TME, immunotherapy, and epigenetic mutations.

In this study, we conducted a thorough pan-cancer multi-omics analysis to examine the molecular correlations between ferroptosis and fatty acid metabolism regulators in 33 cancer types. By performing unsupervised clustering, we identified three distinct clusters related to ferroptosis and fatty acid metabolism based on the TME, gene expression, and biological functions. Utilizing a robust combination of 117 machine-learning algorithms, we developed the FeFAMscore, which demonstrated superior predictive performance in both the training cohort and four independent external validation cohorts. It also effectively exhibits potential in forecasting immunotherapy and chemotherapy drug sensitivity in CRC patients. Overall, the FeFAMscore is promising for the advancement of novel treatment strategies, fostering a nuanced and personalized approach to medicine.

## 2 Methods

### 2.1 Data acquisition and pre-processing

The workflow is illustrated in **Supplementary Figure S1**. The Gene Expression Omnibus (GEO) (https://www.ncbi.nlm.nih.gov/geo/) and The Cancer Genome Atlas (TCGA) (https://portal.gdc.cancer.gov/databases were used to obtain the CRC RNA expression profiles, in addition to the associated comprehensive clinical annotations, including TCGA-COAD, TCGA-READ, GSE17536, GSE17537, GSE29621, GSE38832, and GSE39582. The Meta-cohort and Train cohorts (TCGA-COAD, TCGA-READ, and GSE39582) were established and the batch effects were estimated using the “sva” package in R software. Additionally, three immunotherapy cohorts with different immunotherapy efficacies downloaded from the TIGER website (http://tiger.canceromics.org/), including IMVigor210 (anti-PD-L1), Braun (anti-PD-1), and PRJNA23709 (anti-PD-1 + anati-CTLA4) were investigated. The microarray data from the GEO were normalized and corrected background by the “impute” R package. The ferroptosis regulators and fatty acid metabolism-related genes investigated in this study were extracted from FerrDb (22) and MSigDB (https://www.gsea-msigdb.org/gsea/msigdb/) (**Supplementary Table S1**). Finally, 1448 patients with survival information were acquired from the database. The data of Copy Number Variation (CNV) is extracted from TCGA database in 33 cancers and analyzed by the “matfool” packages.

### 2.2 Unsupervised clustering of ferroptosis regulators and fatty acid metabolism-related genes

The tumor-related FeFAM genes were obtained from TCGA database using “limma” and “survival” packages in the R software. Univariate Cox analysis was used to filter the 50 prognosis genes in the training cohort based on p<0.05. Next, the training cohort was subjected to unsupervised clustering to identify distinct patterns. The potential groupings were delineated using K-means clustering analysis with varying cluster numbers (k = 2–9) (23). We then performed the “Nuclst” package to verify the most appropriate clusters with 28 criteria and repeated 1000 times on resample rate of 0.8 to validate the classification stability. Principal component analysis (PCA) was subsequently employed to validate the clustering results using the expression profiles of these genes. This analysis confirmed the co-expression patterns of ferroptosis regulators and fatty acid metabolism-associated genes.

### 2.3 Cell infiltration estimation

We evaluated the immune cell microenvironment using the CIBERSORT algorithm, EPIC algorithm, MCPCOUNTER algorithm, TIMER algorithm, quantiseq algorithm, and XCELL algorithm of the “IBOR” and “GSVA” R package. Single-sample gene set enrichment analysis (ssGSEA) algorithm was used to verify the results. Additionally, the TIDE algorithm (http://tide.dfci.harvard.edu/) was used to evaluate the tumor immune dysfunction and exclusion (TIDE) score, CAF, dysfunction and exclusion of immune cells, PD-L1, and cytotoxic T cells (CTL) score. A high TIDE score may reflect poor ICI efficacy.

### 2.4 Pathway enrichment analysis

To investigated the biological difference between three patterns and cancer-related pathways, we downloaded “h.all.v7.5.1.symbols.gmt” and “c2.cp.kegg.v7.4.symbols” from the MsigDB database (c2.cp.kegg.symbols), and analyzed using the GSVA program. We further explored the differences in cancer-, immune-, and metabolism-related patterns as reported previously (24–27). The pathways with the highest expression among the three patterns with p<0.05 were considered activated.

### 2.5 FeFAMscore prognostic model construction

To further explore the biofunction and prognostic value of FeFAM genes, we first randomly combined 10 machine learning algorithms, including random survival forest (RSF), elastic network (Enet), Ridge, Stepwise Cox, Lasso, CoxBoost, partial least squares regression for Cox (plsRcox), generalized boosted regression modelling (GBM), supervised principal components (SuperPC), and survival support vector machine (survival-SVM), as reported previously (28). Then, the training cohorts were input as the training group to the combined 117 algorithms, and each model was detected in four independent datasets (GSE17536, GSE17537, GSE29621, and GSE38832). Next, Harrell’s concordance index (C-index) was calculated for each model across all validation cohorts using the FeFAMscore derived from the training cohorts. Based on the average C-index in all validation cohorts, we selected the optimal model and compared the FeFAMscore with those of 69 published models in the past decade, which proved its reliable and robust predictive power.

### 2.6 Cell culture

Normal colon mucosal epithelial cells (NCM460) and HCT116, DLD-1, and CACO2 cell lines were obtained from the Chinese Academy of Sciences (Shanghai, China), cultured in DMEM supplemented with 10% fetal bovine serum (both from Thermo Fisher Scientific, Waltham, MA, USA), and maintained under standard cell culture conditions (37°C, 5% CO_2_) in a cell incubator.

### 2.7 RNA extraction and RT-qPCR

Cellular and tissue RNA was extracted using TRIzol reagent (R411-01, Vazyme, Nanjing, China), followed by reverse transcription using HiScript III RT SuperMix (R323, Vazyme). Quantitative PCR analysis was performed using the Universal SYBR Green Fast qPCR Mix (ABclonal, Hong Kong, China, RK21203). The data were analyzed using the 2(−ΔΔCt) method, with GADPH serving as the internal control. The primer sequences are provided in **Supplementary Table S1**.

### 2.8 siRNA transfection

siRNA-ACAA2-1 or siRNA-ACAA2 (GenePharma, Shanghai, China) was used to silence the ACAA2 gene. The siRNA sequences were as follows: si-ACAA2-1 (sense: 5′-UGCUGAGACAGUGAUUGUATT-3′; antisense: 5′-UACAAUCACUGUCUCUCATT-3′), and si-ACAA2-2 (sense: 5′-GGGCACTGAAGAAAGCAGGA-3′; antisense: 5′-CGTGAACCAGGTGTGCAGTA-3′). Transfection was performed using Lipofectamine 3000 (Thermo Fisher Scientific) following the manufacturer’s instructions.

### 2.9 Cell viability assay

CRC cell viability was evaluated using the Cell Counting Kit 8 (CCK-8, Dojindo, Japan). Briefly, 3000–5000 cells were seeded per well in 96-well plates. Subsequently, 100 μL medium containing 10 μL CCK-8 solution was added to each well and incubated at 37°C for 3 h. The absorbance at 450 nm was measured.

### 2.10 Transwell assay

HCT116 and CACO2 cell lines transfected with siRNAs targeting ACAA2 (si-ACAA2-1 and si-ACAA2-2) or non-targeting control siRNA (si-NC) were harvested, washed twice with PBS, and resuspended in DMEM. The suspended cells were then placed in the upper chamber of 24-well chambers equipped with 8 μm pore inserts.

### 2.11 Colony formation assay

To evaluate colony formation in the monolayer culture, 1000 cells were seeded in 6-well plates. Following two weeks of culture, the colonies were fixed and stained with 4% paraformaldehyde and 0.1% crystal violet for 30 min at room temperature.

### 2.12 Western blot

Protein concentration was determined using the BCA Protein Assay Kit (Thermo Fisher Scientific, USA). Samples containing 30 μg of protein were separated on a 12% SDS-PAGE gel and transferred onto a PVDF membrane. The membrane was blocked with 5% BSA for 2 hours and then incubated overnight at 4°C with the primary antibody. Afterward, the membranes were incubated for 1 hour with the secondary antibody and washed three times with TBST buffer. Antibody signals were detected using the ECL system (Bio-Rad, California, USA).

### 2.13 Immunotherapeutic response prediction

We predicted the immunotherapy response of the FeFAMscore by analyzing the expression of tumor mutational burden (TMB), TIDE score, and differences in pathway enrichment. Based on these results, we calculated the FeFAMscore of patients in the training cohort to explore the function of the FeFAMscore in immunotherapy. Subsequently, we used Subclass Mapping (Submap) to determine the relationship between high or low FeFAMscore groups and anti-PD-1 and anti-CTLA4 antibodies. In addition, we utilized immunotherapy cohorts with clinical response information to validate the immunotherapy response. The IMVigor210CoreBiologies R package was used to obtain transcriptome, survival, and immunotherapy efficacy data for the IMVigor210 cohort (29). The anti-PD-1 and anti-CTLA4 cohorts were validated using Braun and PRJNA23709.

### 2.14 Chemotherapeutic sensitivity prediction

The correlation with FeFAMscore and drug sensitivity was predicted by the GDSC and CTRP datasets with “oncoPredict” packages. The relation with gene expression and drug sensitivity was measured by the “Hmsic” package in R software.

### 2.15 Statistical analysis

Data processing and visualization were performed using R software (version 4.3.2) and GraphPad Prism 8.0, respectively. Group comparisons were performed using the Wilcoxon test for pairwise comparisons, while ANOVA and Kruskal–Wallis tests were used to assess variable distributions among multiple groups, considering normality assumptions. Categorical variables were analyzed using the chi-square and Fisher’s exact tests. Correlations were determined using the Spearman and Pearson techniques. Survival disparities were evaluated using the Kaplan–Meier method and log-rank test. Statistical significance was set at p<0.05, and all p-values were two-tailed.

## 3 Result

### 3.1 Identification of novel correlations between ferroptosis and fatty acid metabolism regulators

**Figure 1:**
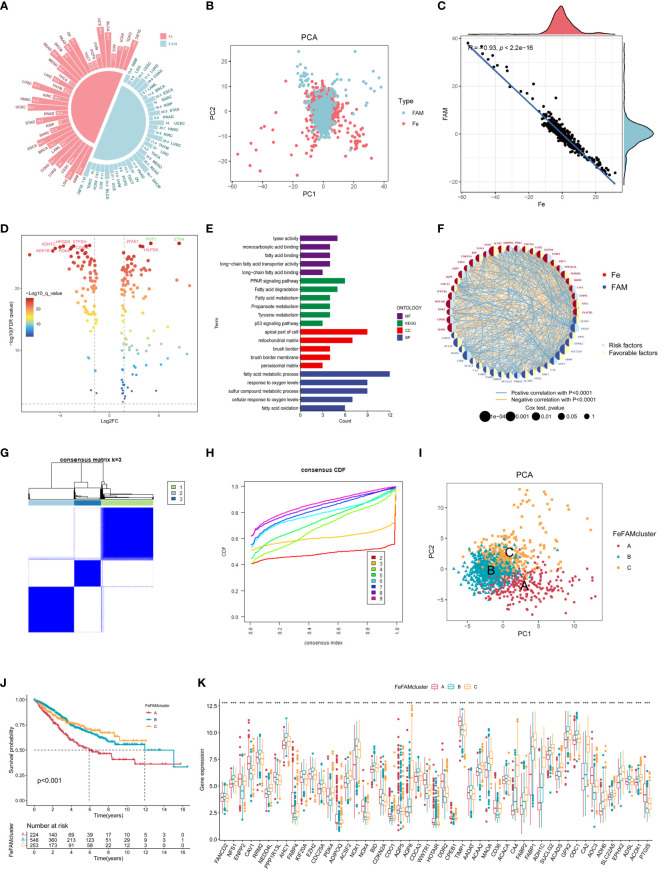
Landscape of genetic and relation of FeFAM regulators and discovery of novel FeFAM patterns. **(A)** Mutation frequency of FeFAM regulators in 33 types cancers in TCGA. **(B)** PCA of FeFAM regulators in the CRC. **(C)** Scatter plot showed the spearmen correlation of FeFAM regulators. **(D)** Volcano plot showed the differential FeFAM regulators in CRC. **(E)** KEGG and GO analyze of 50 OS-related FeFAM regulators. **(F)** Network showed the interactions among FeFAM regulators in CRC. **(G)** A The consensus score matrix of all samples when k = 3. **(H)** The CDF curves of consensus matrix for each k (indicated by colors). **(I)** Principal component (PC) analysis revealed remarkable difference between three FeFAM patterns from train cohort (n = 1029). **(J)** Kaplan-Meier curves of survival for three FeFAM patterns based on CRC patients from train cohort. **(K)** This boxplot demonstrates the expression variations in the FeFAM-related genes among three FeFAM patterns. The top portion represented Fisher’s precise test. The lower portion indicated the Wilcoxon rank-sum test. ***p < 0.001. FeFAM, ferroptosis and fatty acid metabolism regulators.

To explore the relationship between cell death and metabolism, we investigated the crosstalk between ferroptosis-associated regulators and fatty acid metabolism-regulating genes. The 486 ferroptosis-associated genes derived from the FerrDb included markers, suppressors, and drivers (**Supplementary Table S1**). Meanwhile, 272 FAM genes were retained from the MSigDB database. Genome-wide omics data for 33 cancer types were retrieved from the TCGA database for analysis. The frequency of mutations in these genes was significantly correlated between ferroptosis and fatty acid metabolism in the tumors (**Figure 1A**). PCA was performed to measure the levels of ferroptosis-associated and FAM genes in the 33 cancer types database, then the Spearman’s analysis further demonstrated a significant correlation between them (**Supplementary Figure S1A**). Interestingly, the COREAD database, with the largest number of patients, exhibiting a prominent association between them (R = -0.93) (**Figures 1B, C**). Consequently, to further explore the colorectal cancer, the top 10 mutations in ferroptosis-associated regulators and fatty acid genes were identified in 480 (95.98%) of 497 patients with COREAD. The highest mutation frequencies were detected in TP53 (67%), KRAS (44%), and PIK3CA (26%) (**Supplementary Figure S2B**). The exploration of copy number variation (CNV) alteration frequency showed a high incidence of CNV gains in the TCGA cohort, demonstrating the potential for therapy in CRC (**Supplementary Figure S2C**). The locations of FeFAM genes with CNVs on the chromosomes are marked in the Circle Map (**Supplementary Figure S2C**). Additionally, co-mutations were common among these genes (**Supplementary Figures S2E, F**).

Based on the analysis of results, we compared the CRC and normal samples from COREAD databases in TCGA, which finally identified 159 ferroptosis-associated and fatty acid metabolism-related genes according to logFC>1 and FDR <0.05 (**Figure 1D**). To counterbalance the implications between TCGA and GEO database, we enrolled COREAD database and GSE39582 and adopted the “sva” package to remove batch effects and extract relevant genes as the training cohort. A total of 50 genes were subsequently screened using univariate Cox regression analysis (P <0.05) of the FeFAM genes in the combined database (**Supplementary Figure S2G**). The KEGG analysis, depicted using a barplot, revealed enrichment of these genes in pathways such as “PPAR signaling pathway”, “fatty acid degradation”, “fatty acid metabolism”, “propanoate metabolism”, “tyrosine metabolism”, and “p53 signaling pathway”. Furthermore, GO analysis of molecular functions (MF), biological processes (BP), and cellular components (CC) highlighted their relevance in fatty acid metabolism, response to oxygen levels, and cancer pathways. (**Figure 1E**). Network analysis offered a holistic view of the prognostic implications and molecular interactions within the FeFAM framework (**Figure 1F**). Considering the discernible differences in the transcriptional profiles and the unique interplay between these molecules, dysregulation within the FeFAM network significantly contributes to CRC initiation and progression.

### 3.2 Discovery of novel FeFAM patterns through unsupervised clustering analysis

To elucidate the potential FeFAM phenotypes in CRC, we utilized K-means-based unsupervised clustering in the training cohort. The R package “ConsensusClusterPlus” was used to initially categorize the patients with CRC into k (k=2–9) FeFAM clusters (**Figure 1G**; **Supplementary Figures S3A-K**). The cumulative distribution function (CDF) curves, derived from the consensus score matrix and PAC statistics, elucidated the optimal number of clusters (k=3) across the entire training patient cohort. These clusters, denoted A, B, and C, exhibited discernible segregation patterns (**Figure 1H**). Nbclust testing, which included 28 criteria, yielded the same results (**Supplementary Figure S3L**). The PCA demonstrated a clear distinction between the three clusters (**Figure 1I**). The Kaplan–Meier curve showed that cluster C had better survival prognosis than clusters A and B (p<0.001) (**Figure 1J**). The expression of FeFAM genes also indicated the ability to differentiate between the three subtypes (**Figure 1K**).

### 3.3 TME characterization in different FeFAM patterns

**Figure 2:**
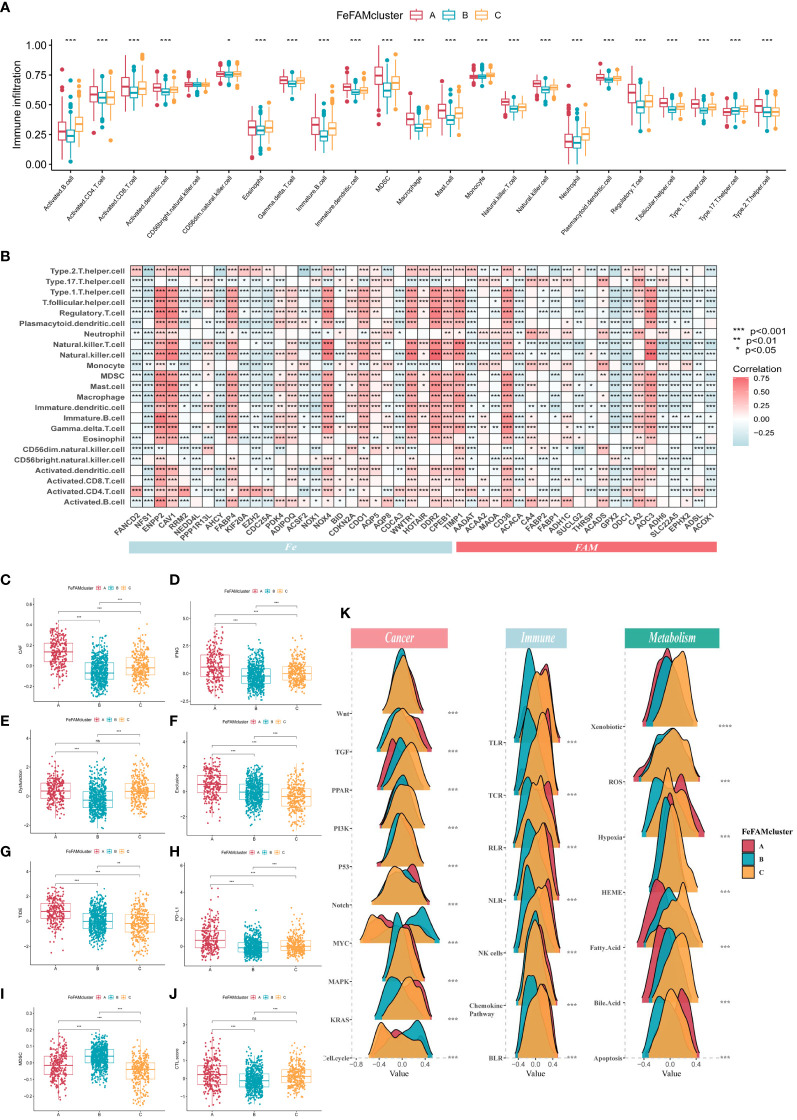
Characterization of tumor microenvironment, signing and immune pathways in different FeFAM patterns. **(A)** Characteristics of immune infiltrating cells in different FeFAMclusters. **(B)** Characteristics of immune infiltrating cells in different FeFAM regulators. **(C-J)** Box plots showed the significant difference in CAF **(C)**, IFNG **(D)**, Dyfunction **(E)**, Exclusion **(F)**, TIDE **(G)**, PD-L1 **(H)**, MDSC **(I)**, and CTL.score **(J)**. **(K)** The cancer-related, immune-related and metabolism-related pathways between the three FeFAM subtypes. * p < 0.05, ** p < 0.01, *** p < 0.001, ns, not significant. FeFAM, ferroptosis and fatty acid metabolism regulators.

The ssGSEA method, which simulates the entire tumor immune process, was first used to calculate tumor immune cell infiltration in the training cohorts to investigate the differences in the TME (**Figure 2A**). Next, six different algorithms, such as CIBERSORT, EPIC, MCPCOUNTER, TIMER, QuantiSeq, and XCELL, obtained the same results, verifying the crucial effects of FeFAM genes in the immune system (**Supplementary Figure S4A**). Meanwhile, according to the Spearman’s correlation analysis, almost all FeFAM genes were significantly implicated in the immune microenvironment composition (**Figure 2B**). Ferroptosis-associated regulators, including ENPP2, CAV1, FABP4, PDK4, ADIPOQ, NOX4, COKN2A, CDO1, WWTR1, DDR2, CPEB1, and TIMP1, are preferentially associated with the most immunosuppressive cells, whereas FAM genes correlated with immune microenvironment activation. Furthermore, Spearman’s analysis demonstrated significant co-expression of the Fe and FAM genes (**Supplementary Figure S4B**). Compared to FeFAM clusters B and C, FeFAM cluster A had a significantly worse prognosis. Analysis of gene signatures revealed an increased presence of immune cells exhibiting notable immunosuppressive functionality, macrophages, regulatory T cells (Tregs), and type 2 T helper cells, including within FeFAMA cluster A across all cohorts. Remarkably, CD4+ T cells, CD8+ T cells, neutrophils, dendritic cells, and natural killer (NK) cells were abundant in the FeFAM cluster A across nearly all the algorithms, suggesting that the immune cells within FeFAM cluster A may concurrently govern immune evasion and anti-tumor activities. Despite having similar prognoses, FeFAM clusters B and C exhibited contrasting levels of immune infiltration, implying that they may have different immunotherapeutic potentials. The XCELL and ESTIMATE algorithms also demonstrated high immune, stroma, and microenvironment scores in FeFAM clusters A and C (**Supplementary Figure S4C**). Consequently, FeFAM clusters A, B, and C were considered immune-excluded, immune-desert, and immune-activated clusters, respectively. To ensure stability of the results, the TIDE algorithm, which is commonly adopted to measure immune escape levels and ICD treatment efficacy, was used. We discovered that FeFAM cluster A showed the highest CAF, IFN, TIDE, PD-L1 and exclusion among the three clusters, consistent with the above analysis results and possibly attributed to immune escape (**Figures 2C–J**). Interestingly, although FeFAM cluster A had more MDSCs than FeFAM cluster C, their CTL.scores and dysfunction were similar, suggesting that activated ferroptosis and fatty acid pathways may inhibit intratumoral CD8+ T cell effector function and impair their anti-tumor ability, which was similar to previously reported results (12, 30–32). Above all, ferroptosis regulators may cooperate with fatty acid metabolism-associated genes to contribute to a particular immune microenvironment, thereby presenting potential targets for immunotherapy.

### 3.4 Signaling and immune pathway differences between the FeFAM patterns

Utilizing the “gsva” package, we executed GSVA-enrichment experiments to investigate various cancer-related signaling pathways across the three patterns within the Hallmarker and KEGG pathways (**Supplementary Figure S4D**). The findings indicate that FeFAM cluster A was significantly enriched in immune- and tumor-related pathways, such as “apoptosis”, “epithelial mesenchymal transition (EMT)”, “inflammatory response”, and “VEGF signaling pathway”. The FeFAM clusters B and C are two distinct groups with specific associations in terms of their biological functions and metabolic pathways. FeFAM cluster B, for instance, is substantially associated with “DNA repair and replication”, “protein export”, and “spliceosome”, while FeFAM cluster C is associated with the metabolic pathway “fatty acid metabolism”, “linoleic acid metabolism”, “nicotinate and nicotinamide metabolism” and “nitrogen metabolism”. Consequently, we further investigated the carcinogen-signaling, immune-related, and metabolic pathways to compare the differences among the three patterns. Wnt, TGF, Notch, MAPK, KRAS, TLR, TCR, RLR, NK cells, chemokine pathway, hypoxia, and apoptosis were activated in cluster A. PPAR, PI3K, P53, xenobiotics, ROS, HEME, fatty acids, and bile acids were activated in cluster C, indicating that ferroptosis and fatty acid metabolism may be upgraded to improve prognosis and prevent immune escape (**Figure 2K**). These analyses provided additional evidence that FeFAM molecules regulate the immune microenvironment and facilitate immune evasion in patients with CRC through diverse signaling pathways. This underscores the potential of FeFAM as a promising target for immunotherapy.

### 3.5 Integrated construction and consistent prognostic value of the FeFAMscore

**Figure 3:**
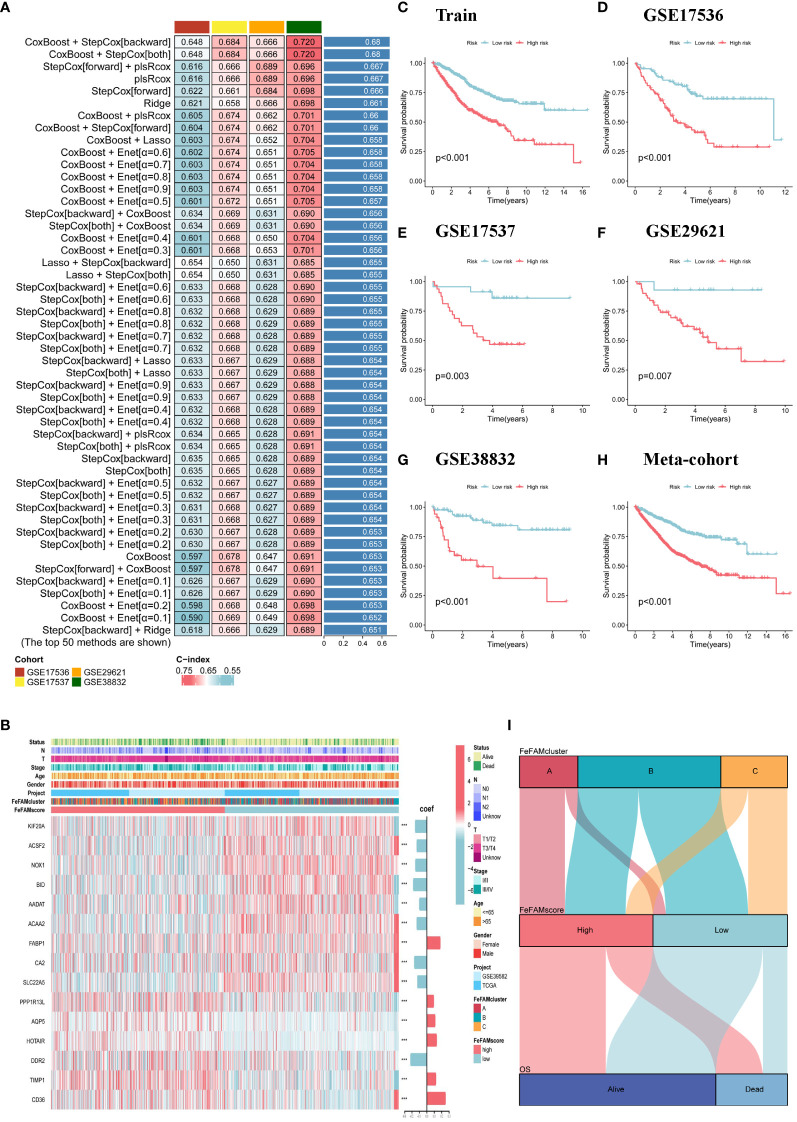
Construction of a machine learning-based signature. **(A)** The top C-index of 50 machine learning methods in four validation cohorts. **(B)** The heatmap demonstrates the relationships between the three FeFAM phenotypes, clinicopathologic characteristics, coef value and the expression variations of the FeFAM-related genes in train cohort. **(C–H)** Kaplan-Meier curves of OS according to the FeFAMscore in Train cohorts (log-rank test: p<0.001) **(C)**. GSE17536 (Log-rank test: p<0.001) **(D)**. GSE17537 (Log-rank test: p = 0.003) **(E)**. GSE29621 (Log-rank test: p = 0.007) **(F)**. GSE38832 (Log-rank test: p < 0.001) **(G)**. Meta-cohort (Log-rank test: p < 0.001) **(H)**. **(I)** Alluvial diagram showing the correlation of FeFAMclusters and FeFAMscore. *** p < 0.001. FeFAM, ferroptosis and fatty acid metabolism regulators.

Based on the varying expression levels of FeFAM genes among the three patterns, we subjected the 50 FeFAM-related genes to our machine learning-based integrative approach to construct an FeFAM-related signature, termed the FeFAMscore. In the training cohort, 117 algorithms generated from a random permutation of 10 machine-learning algorithms were employed to compute the C-index using a 10-fold cross-validation framework. The model was subsequently evaluated across four cohorts to gauge its predictive efficacy and to determine its consistency across different datasets. Following this evaluation, the model with the highest average C-index among the validation cohorts was identified. Specifically, the combination of CoxBoost and StepCox (backward and both) yielded the highest average C-index of 0.689, establishing it as the optimal model (**Figure 3A**; **Supplementary Table S2**). Fifteen genes were first screened using the CoxBoost model and then subjected to backward Cox proportional hazards regression. A final set of 15 genes was identified, including KIF20A, ACSF2, NOX1, BID, AADAT, ACAA2, FABP1, CA2, SLC22A5, PPP1R13L, AQP5, HOTAIR, DDR2, TIMP1, and CD36 (**Figure 3B**). Subsequently, the FeFAM score for each patient was determined by employing the expression levels of 15 genes, which were weighted using the regression coefficients obtained from a Cox model (**Figure 3B**). Subsequently, all patients were dichotomized into high- and low-FeFAMScore groups. These 15 genes significantly distinguished high-risk individuals from low-risk individuals (**Figures 3C–H**). Patients categorized into the high FeFAMscore group exhibited significantly poorer overall survival (OS) compared to those in the low FeFAMscore group, as determined by Kaplan–Meier survival analysis in both the combined training (N=1029, P<0.001) and four validation datasets: GSE17536 (N=177, P<0.001), GSE17537 (N=55, P=0.003), GSE29621 (N=65, P=0.007), and GSE38832 (N=122, P<0.001). A similar outcome was observed in the meta-cohort (N=1448), thereby affirming the predictive accuracy and reliability of the model. An alluvial diagram illustrates the correlation between FeFAMcluster and FeFAMscore (**Figure 3I**).

### 3.6 Consistent prognostic value of FeFAMscore

**Figure 4:**
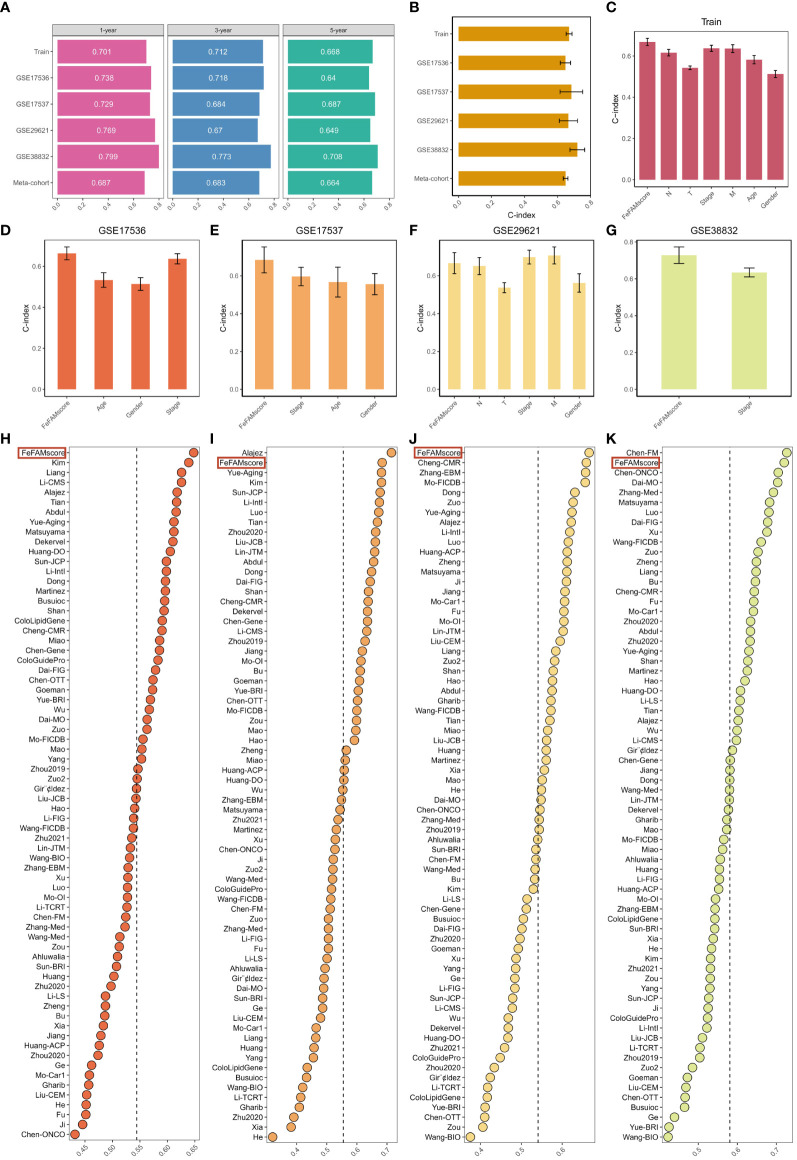
Comparison between the FeFAMscore and the other 69 signatures in colorectal cancer. **(A)** Time-dependent ROC analysis for predicting OS at 1,3, and 5 years in train cohort (n = 1029), GSE17536 (n = 177), GSE17537 (n = 55), GSE29621 (n = 65), and GSE38832 (n = 122). **(B)** C-index of FeFAMscore across all datasets. **(C-G)** The performance of FeFAMscore was compared with other clinical variables in predicting prognosis. Train **(C)**, GSE17536 **(D)**, GSE17537 **(E)**, GSE29621 **(F)**, and GSE38832 **(G)**. **(H-K)** C-index of FeFAMscore and 69 published signatures in GSE17536 **(H)**, GSE17537 **(I)**, GSE29621 **(J)**, and GSE38832 **(K)**. *** p < 0.001. FeFAM, ferroptosis and fatty acid metabolism regulators.

Receiver operating characteristic (ROC) curve analysis was conducted to evaluate the discriminative ability of the FeFAMscores. In the training cohort, the areas under the ROC curve (AUC) for 1-, 3-, and 5-year survival were 0.701, 0.712, and 0.668, respectively. Furthermore, excellent results were also indicated in the test cohorts, including 0.738, 0.718, and 0.64 in GSE17536; 0.729, 0.684, and 0.687 in GSE17537; 0.769, 0.670, and 0.649 in GSE29621; and 0.799, 0.773, and 0.708 in GSE38832 (**Figure 4A**). The meta-cohort of these patients showed AUC values of 0.687, 0.683, and 0.644, indicating that the FeFAMscore model is predictive and reliable in multiple independent CRC cohorts (**Figure 4A**).

The C-index [95% confidence interval] was 0.67 [0.652–0.688], 0.648 [0.617–0.679], 0.684 [0.639–0.807], 0.666 [0.614–0.790], 0.720 [0.649–0.804], 0.649 [0.646–0.711] in the four independent validation cohorts and meta-cohorts, respectively (**Figure 4B**). To predict patient prognosis, clinical characteristics including age, sex, T stage, N stage, M stage, and stage are widely acknowledged. Therefore, the C-index was applied to measure the predictive accuracy between the FeFAMscore and clinical traits in the training and four independent validation cohorts. The FeFAMscore exhibited significantly higher predictive accuracy than other clinical traits in the training, GSE17536, GSE17537, and GSE38832 cohorts (**Figures 4C–G**). In contrast, the performance of the FeFAMscore in GSE29621 cohort was similar to that of the M stage and stages, which may have been due to the small sample size and data bias. These results indicate that FeFAMScore may be a prospective alternative biomarker for predicting survival risk in clinical practice.

### 3.7 Resilient predictive performance of FeFAMscore

As the sequencing depth continually increases, CRC treatment outcomes are well predicted. Machine learning-based prognostic models for CRC have been increasingly developed in recent years. To quantify the performance of the FeFAMscore, we systematically retrieved mRNA signatures from CRC research over the past decade and finally acquired 69 mRNA signatures. We compared the predictive ability of the FeFAMscore using the C-index value in the four independent validation cohorts. The FeFAMscore ranked first in the GSE17536 and GSE29621 datasets, followed by GSE17537 and GSE38832 (**Figures 4D-G**). However, some models exhibited appreciable predictive performance for the GSE17537 and GSE38832 datasets and performed moderately in other cohorts, further proving the uniqueness and reliability of our models. The Chen-FM model, for instance, showed a better C-index than the FeFAMscore in GSE38832 and was poorly displayed in GSE17536, GSE17537, and GSE38832 with a C-index of less than 0.6. The above results demonstrated the good predictive performance of the FeFAMscore (**Figures 4H–K**).

### 3.8 ACAA2 is associated with tumor progression in CRC

**Figure 5:**
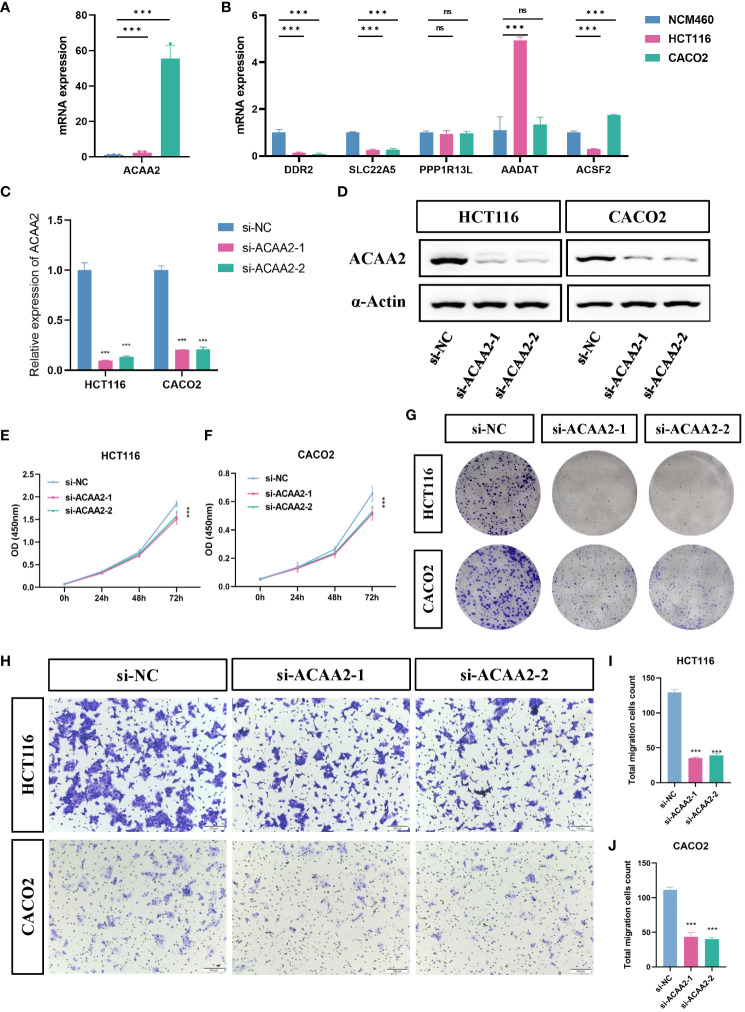
ACAA2 promotes colorectal cancer progression. **(A, B)** Comparison of the expression levels of ACAA2, DDR2, SLC22A5, PPP1R13L, AADAT and ACSF2 between NCM460 cells, HCT116 cells, and CACO2 cells. **(C)** The knockdown efficiency of ACAA2 in HCT116 cells and CACO2. **(D)** Representative western blots examined the expression of ACAA2 protein levels after the downregulation of ACAA2 of HCT116 and CACO2 cell lines. **(E, F)** The CCK8 assay detected cell viability after decreased ACAA2 expression in HCT116 **(E)** and CACO2 **(F)** cell lines. **(G)** Knockdown of ACAA2 significantly reduced the number of clones in HCT116 and CACO2 cell lines. **(H)** The transwell assay detected the migration ability of HCT116 and CACO2 cells after decreased ACAA2 expression. **(I-J)** Quantification results of numbers of relative migration rates in transwell assay in HCT116 **(I)** and CACO2 **(J)** cells. *** p < 0.001, ns, not significant.

To further evaluate the expression and function of the FeFAMscore, we first performed RT-qPCR in cell lines from patients with CRC for the six genes. The other nine genes involved in the FeFAM score have been demonstrated by other researchers (33–41). Compared with those in normal human colonic cells (NCM460 cells), the expression of ACAA2 was significantly higher in HCT116 and CACO2 cells, while the expression of ACSF2, DDR2 and SLC22A5 was significantly increased in the CRC cells (**Figures 5A, B**). Among the expression and correlation of the genes, ACAA2 was significantly overexpressed in the tumor tissues and strong correlated with ferroptosis regulators. We then used two small interfering RNAs (siRNAs) to downregulate ACAA2 expression in HCT116 and CACO2 CRC cell lines (**Figure 5C**). The western blot further demonstrated the results (**Figure 5D**). Cell viability was reduced by ACAA2 downregulation after 72 h (**Figures 5E, F**). In addition, Cell colony formation experiments demonstrated a significant reduction in colony numbers in HCT116 and CACO2 cell lines following ACAA2 knockdown. (**Figure 5G**). Transwell assays also confirmed that ACAA2 knockdown significantly reduced the migratory ability of CRC cells (**Figures 5H–J**). Taken together, these results indicate that ACAA2 not only induces CRC cell proliferation, but also promotes CRC cell migration.

### 3.9 Mutation status in high and low FeFAMscore groups

**Figure 6:**
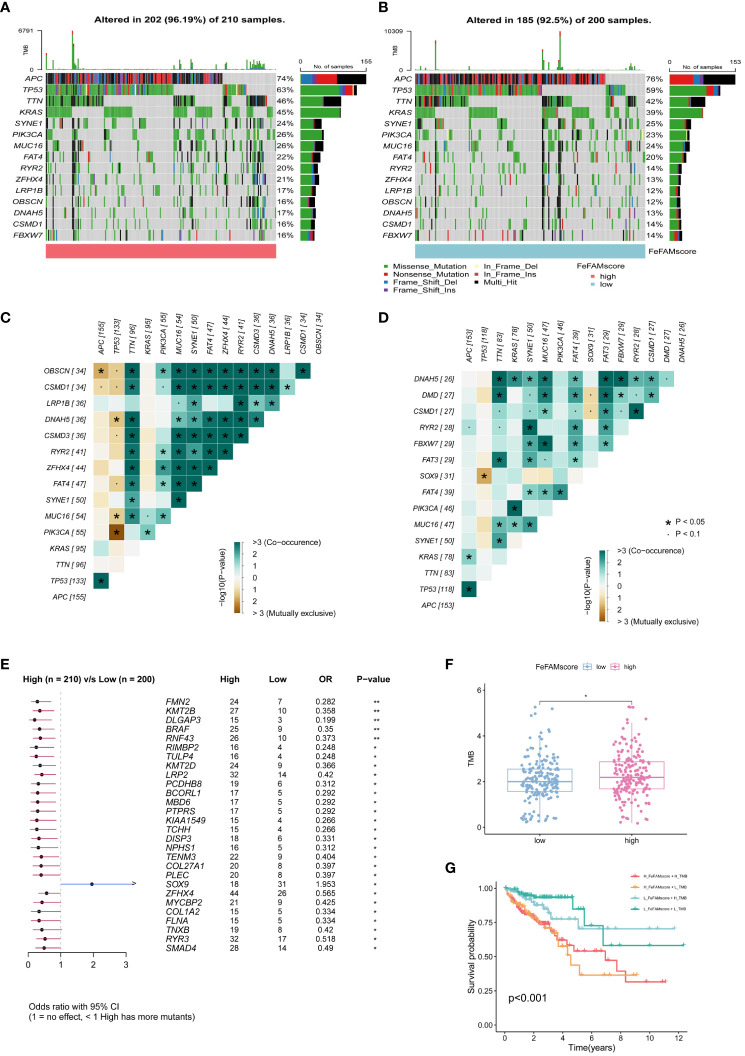
The FeFAMscore related to the tumor mutation status. **(A, B)** Visual summary showing common genetic alterations in low **(A)** and high **(B)** FeFAMscore groups. **(C, D)** Interaction effect of genes mutating in the low **(C)** and high **(D)** FeFAMscore groups. **(E)** Forest plot gene mutations in the CRC patients. **(F)** The TMB in low and high FeFAMscore groups. **(G)** Survival analysis for CRC patients measured by both FeFAMscore and TMB using Kaplan-Meier curves. * p < 0.05, ns, not significant. FeFAM, ferroptosis and fatty acid metabolism regulators.

To explore the mechanisms underlying the FeFAMScore, somatic mutations in the patients with CRC in the TCGA cohort were further analyzed. As expected, more mutations in top 15 genes were observed in the high FeFAMscore group than that in the low FeFAMscore group (**Figures 6A, B**). In addition, co-occurrence and mutual exclusion were observed among these genes (**Figures 6C, D**). The forest plot also revealed that the BRAF gene, which is generally regarded as a potential prognostic risk factor, had more mutations than the low one, which indicates poor prognostic survival and worse ICI efficacy (**Figure 6E**). Moreover, the high FeFAMscore groups exhibited a higher TMB than the low FeFAMscore groups (**Figure 6F**). Poor prognosis was also demonstrated by high TMB combined with a high FeFAMscore (**Figure 6G**).

### 3.10 Immune characteristics related to FeFAMscore

We first adopted the ssGSEA algorithm to explore the correlation between tumor-infiltrating immune cells and the FeFAMscore in the training cohorts (**Supplementary Figure S5A**), which indicated that the FeFAMscore had a positive relationship with the immune cells. These findings suggest that the high FeFAMscore group, despite exhibiting a worse prognosis, harbored a higher abundance of immunologically activated cells than the low FeFAMscore group. Additionally, the high FeFAMscore group demonstrated an increased presence of immunosuppressive cells such as MDSCs, macrophages, mast cells, and regulatory T cells (**Supplementary Figure S5B**). Therefore, ssGSEA and six external algorithms, including CIBERSORT, EPIC, MCPCOUNTER, TIMER, quantiseq, and XCELL were further used, yielding similar results: the high FeFAMscore group had a high ImmuneScore, StromaScore, and MicroenvironmentScore (**Supplementary Figure S5C**). Next, we investigated the cancer-, immune-, and metabolic-related pathways between the two groups, which means that a high FeFAMscore prefers to be enriched in cancer-related and immune-related pathways (**Supplementary Figure S5D**). Interestingly, fatty acid metabolism was significantly activated in the low FeFAMscore group, which may improve the prognosis. Based on these findings, the high FeFAMscore groups probably had several targets that may benefit from specifically targeted immunotherapy, even though they had a worse prognosis.

### 3.11 FeFAMscore predicts CRC response to immunotherapy

**Figure 7:**
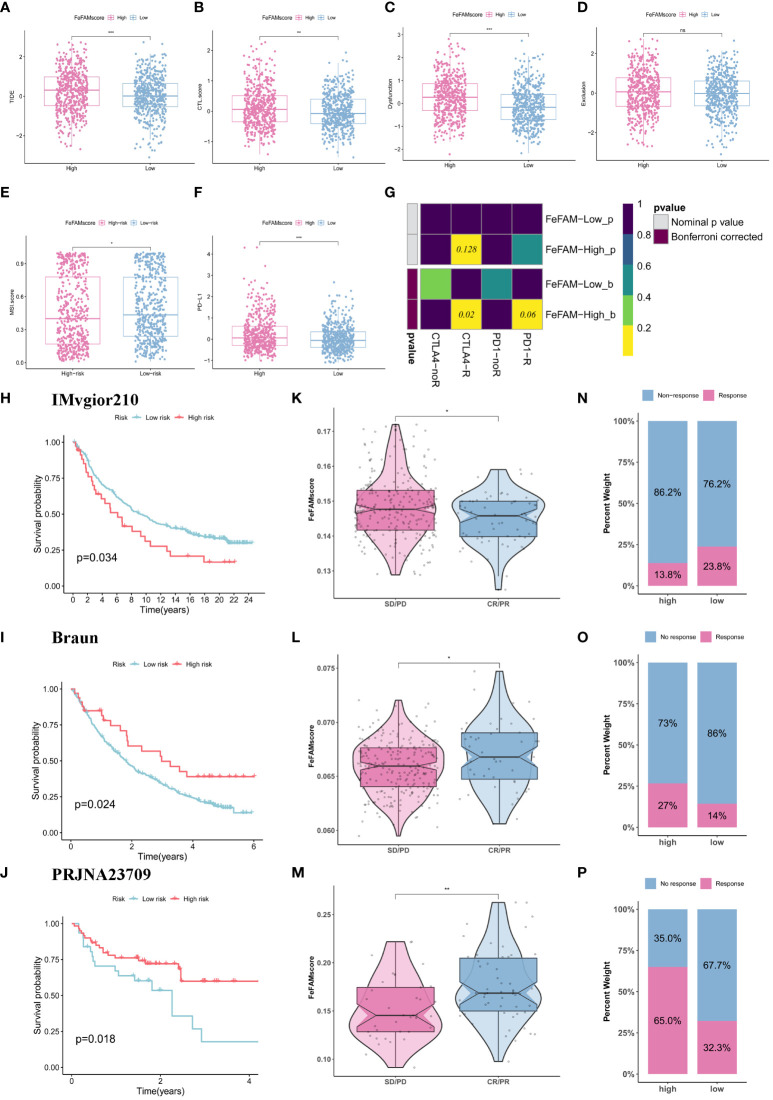
FeFAMscore predicts the response of colorectal to immunotherapy. **(A-F)** Box plots showed the significant difference in TIDE **(A)**, CTL.score **(B)**, Dyfunction **(C)**, Exclusion **(D)**, MDSC **(E)**, PD-L1 **(F)**. **(G)** The submap algorithm predicts the probability of anti-PD1 and anti-CTLA4 immunotherapy response in high and low FeFAMscore groups. **(H-J)** The Kaplan–Meier curve exhibited a significant difference in survival rate between the high and low FeFAMscore groups in the IMVgior210 cohort **(H)**, Braun **(I)**, and PRJNA23709 **(J)**. **(K-M)** The Wilcoxon rank-sum test of FeFAMscore variation in the IMVgior210 cohort **(K)**, Braun **(L)**, and PRJNA23709 **(M)**. **(N-P)** The stacked histogram shows the difference in immunotherapy responsiveness between the high and low FeFAMscore groups in the IMVgior210 cohort **(N)**, Braun **(O)**, and PRJNA23709 **(P)**. FeFAM, ferroptosis and fatty acid metabolism regulators. * p < 0.05, ** p < 0.01, *** p < 0.001, ns: not significant.

We first used TIDE and ESTIMATE algorithms to measure the microenvironment in patients with low and high FeFAMscores (**Figures 7A–F**; **Supplementary Figure S5E**). The results indicated that the high FeFAMscore group was associated with high immune infiltration but high TIDE, CTL.score, dysfunction, MSI, and PD-L1, which means that although these individuals with poor survival prognosis contain immunosuppressive cells, this is the main reason for immune evasion and poor ICI efficacy in these individuals. This suggests that the high FeFAMscore group with poor survival may be a special target for activated immune cells, improving survival prognosis. Hence, the submap algorithm was used to assess the feasibility of the FeFAMscore in predicting immunotherapy efficacy. These findings affirmed that individuals in the high FeFAMscore group may benefit from both anti-PD-1 and anti-CTLA4 therapies (**Figure 7G**).

Based on the previous analysis, we determined the FeFAMscore in the IMvigor210 cohorts (anti-PD-L1 therapy) (42), Braun cohorts (anti-PD-1 therapy) (43) and PRJNA23709 (anti-PD-1 therapy + anti-CLTA4 therapy) (44). In the IMvigor210 dataset, patients with low FeFAMscores exhibited better prognoses than those with high FeFAMscores. Additionally, individuals with low FeFAMscores were likely to respond favorably to anti-PD-L1 immunotherapy (**Figures 7H, K, N**). Interestingly, in the Braun cohort, patients with high FeFAMscores demonstrated the potential for benefit from anti-PD-1 therapy (**Figures 7I, L, O**). As expected, patients with renal cell carcinoma and high FeFAMscore had significantly improved survival probability and were likely to respond to anti-PD-1 therapy. The results were shown in PRJNA23709 when patients with high FeFAMscore received combined anti-PD-1 and anti-CTLA4 therapy (**Figures 7J, M, P**). They not only greatly improved survival prognosis, but also acquired a remarkable response rate to immune therapy. These results suggest that individuals with a low FeFAMscore may benefit from immunotherapy, but that individuals with a high FeFAMscore obtain excellent response rates and survival with specific immunotherapies.

### 3.12 FeFAMscore predicts CRC sensitivity to chemotherapeutic response analysis

To assess potentially effective drugs associated with the FeFAMscore, we investigated chemotherapeutic agents using the “oncoPredict” package. With the compared differences between two risk FeFAM groups by the Wilcoxion test with p<0.05, we significantly filtered 316 (total: 545) and 95 (total: 224) compounds in the CTRP and GDSC, respectively. Next, we investigated the drug intersections in the two databases (**Supplementary Figure S6A**). Spearman’s method was used to measure the correlation between FeFAM genes and drug sensitivity. Some genes and drugs, such as KIF20A and AADAT, interacted antagonistically (**Supplementary Figures S6B, C**). However, the ACSF2 and FABP1 interacted synergistically. To validate the irreplaceable role of FeFAM molecules in chemotherapy, we applied the FeFAMscore to guide chemotherapeutic selection for CRC in clinical practice. Exploring in the CRC related chemotherapy drugs, the osimertinib, oxaliplatin, gefitinib, eriotinib, navitoclax, and cyclophosphamide are beneficial for the patients with high FeFAMscore, unlike irinotecan, niraparib, gemcitabine, niraparib, dabrafenib, and selumetinib (**Supplementary Figures S6D–O**). These findings underscore the availability of diverse chemotherapy modalities tailored to specific patients with CRC, thereby paving the way for precision chemotherapy and personalized treatment approaches.

## 4 Discussion

Several therapeutic modalities, including surgery, chemotherapy, immunotherapy, radiotherapy, and targeted therapy have emerged as key strategies in CRC research (3, 5, 45, 46). These diverse treatment approaches represent a profound advancement in CRC management, reflecting the multifaceted approach necessitated by disease complexity (47). Among these, immunotherapy is a promising frontier that exploits the intricate interplay between the immune system and malignant cells to elicit therapeutic responses (48). However, a subset of patients with CRC exhibiting deficient mismatch repair or microsatellite instability-high (dMMR/MSI-H) represents a relatively small fraction, comprising approximately 15% and 4% patients with CRC and metastatic colorectal cancer (mCRC), respectively; a proportion of these patients swiftly progress to a state of immune resistance (38, 39). The AJCC staging system is a widely accepted criterion for clinical management and encompasses therapeutic decision-making and surveillance strategies for CRC. The utility of the AJCC staging system is constrained by the variability in clinical outcomes observed among patients classified within the same stage (49). This may not only result in overtreatment and undertreatment, but also make it difficult to reflect the sensitivity of immunotherapy and chemotherapy because it does not reflect the TME. To bridge this gap, identifying novel prognostic and therapeutic targets for CRC is vital.

Cell death is a regulated process in cells and may be related to metabolism during tumor progression, metastasis, and drug resistance. Ferroptosis is an iron-related cell death pathway characterized by lipid peroxide accumulation (50, 51). Fatty acid metabolism plays a pivotal role in tumorigenesis, disease progression, and treatment resistance by facilitating augmented lipid synthesis, storage, and catabolism (52). Numerous studies have demonstrated that ferroptosis is significantly correlated with metabolism, particularly lipid metabolism (50–53). For instance, ASL4, a fatty acid metabolism-related gene, is induced by the T cell-derived interferon (IFN)-γ to change the tumor lipid pattern, which increased arachidonic acid (AA) production to promote ferroptosis (32). SLC47A1, which regulates lipid remodeling and survival during ferroptosis, inhibits the anticancer activity of ferroptosis inducers (54). Moreover, they interact to modulate drug sensitivity (32, 55). Consequently, the influence of ferroptosis regulators and fatty acid molecules on the TME as well as their predictive capacity for prognosis and response to immunotherapy in CRC, remain unclear.

This study elucidated the genetic and transcriptomic diversity of FeFAMs across 33 cancer species using a multi-omics approach. Similar frequencies observed among the ferroptosis and fatty acid metabolism regulators indicate their interconnectedness. Spearman’s rank correlation coefficient further demonstrated a strong correlation of ferroptosis and fatty acid metabolism regulators between 33 cancer species, especially in CRC (R= -0.93; p<0.001). Furthermore, after screening 50 genes using the “limma” package and univariate Cox regression analysis, the patients were stratified into three distinct phenotypes, each exhibiting significant disparities in genetic profiles and immune infiltration within the clusters.

We then distinguished three ferroptosis- and fatty acid metabolism-related patterns, named FeFAM clusters A/B/C. The TME characteristics in the three patterns indicated differential immune cell compositions. FeFAM clusters A and C were correlated with immune cell abundance; however, they displayed disparate prognostic outcomes. The FeFAM cluster A processed with high immune activate, StromaSocre, PD-L1 expression, CAF expression, and high activation of TGF-β signaling pathway proved a strong relation with immune-exclude subtypes, while the FeFAM cluster C associated with excellent prognosis and abundant immune cell was regarded as an immune-inflamed phenotype. Interestingly, FeFAM cluster C exhibited the same levels of dysfunction and CTL.score and high metabolism levels, such as the ROS and fatty acids, than FeFAM cluster A, which indicated that the interaction of ferroptosis and fatty acid metabolism may coordinate with T cell dysfunction (32). MDSC density was the highest in FeFAM cluster B, which is defined as the immune desert subtype. These results demonstrate that FeFAM molecules play a vital role in the TME and may trigger extrinsic immune escape.

Further FeFAM molecule characterization in CRC is imperative. Developing features associated with FeFAM molecules will facilitate prognosis prediction and immune response evaluation in CRC. To avoid model selection bias and confirm model accuracy, we randomly combined 10 classical algorithms and eventually obtained 117 combined algorithms. Subsequently, we developed FeFAMscore, a machine-learning-based FeFAM-related model, which exhibited the best performance among the 117 signatures. Recognizing the heterogeneity often observed in the patients with CRC, we externally validated the FeFAMscore using four additional CRC databases. The highest C-index among the validations not only confirmed the selection of the optimal model, CoxBoost combined with stepwise Cox (backward direction), but also underscored the accuracy and generalizability of the model. Moreover, a comparison of 69 published CRC signatures showed improved accuracy, exhibiting robustness. To validate model accuracy, we identified ACAA2 as a key FeFAMscore regulator and conducted cellular experiments, which revealed that ACAA2 promotes CRC proliferation and migration.

Furthermore, FeFAMscore demonstrated a robust association with survival outcomes. The adverse prognosis observed in the high FeFAMscore group may be attributed to improved activation of anti-immune components, potentially fostering a TME conducive to immune evasion. Interestingly, the FeFAMscore and tumor immune infiltration extent in CRC is positively correlated. Moreover, mutations leading to tumor neoantigens, along with a high tumor mutational burden (TMB), increase tumor immunogenic neoantigen abundance (56). The patients with high TMB may benefit from immunotherapy, but many patients do not achieve the desired results (42). Similar results were observed in this study. Tide, a computational method developed by Peng Jiang, models T cell dysfunction and exclusion mechanisms of tumor immune evasion by infiltration of cytotoxic T lymphocytes (CTL), showed the same results (42). However, owing to the variances in immune-related pathways between the two cohorts and the primary mechanism of immune evasion being dysfunction, it is plausible that there may be specific therapeutic benefits for the high FeFAMscore group. The Submap algorithm further supported these results, showing that the high FeFAMscore group processes were more sensitive to anti-PD-1 and anti-CTLA4 therapies. According to the results of previous studies, the predominant mechanism suggests that both high and low fatty acid metabolism can affect the expression level of iron death, consequently affecting the mode of action of CTLs (30, 32). The high FeFAMscore group exhibited diminished fatty acid metabolism, potentially regulating ferroptosis to augment CTL sensitivity. To validate these results, we analyzed the FeFAMscores in immunotherapy cohorts receiving anti-PD-L1 therapy, anti-PD-1 therapy, and anti-PD-1 combined with anti-CTLA4 therapy. Similar results were observed in these cohorts, further demonstrating the limitations of TMB and TIDE. Furthermore, regarding chemotherapeutic agents, the FeFAMscore exhibited promising predictive capabilities. Collectively, these findings indicate that FeFAMscore holds promise as a valuable tool for formulating efficacious CRC treatment strategies.

This study had some limitations. First, the intricate regulatory mechanisms governing ferroptosis and fatty acid metabolism remain unclear. Moreover, retrospective cohorts sourced from publicly available online databases were used. Large multicenter prospective clinical investigations are warranted to corroborate these findings. Finally, to validate the predictive utility of the FeFAMscore in immunotherapy response, additional indicators are required, along with prospective cohorts of patients with glioma undergoing immunotherapeutic interventions.

In conclusion, through a comprehensive approach integrating multicenter analysis and machine learning algorithms, we developed a stable and reliable prognostic and immunotherapeutic response predictor, the FeFAMscore, for CRC. Notably, the high FeFAMscore group demonstrated an increased sensitivity to anti-PD-1 and anti-CTLA4 therapies. The FeFAMscore holds promise as a valuable tool for tailoring efficacious treatment regimens for CRC.

## Data availability statement

The datasets presented in this study can be found in online repositories. The names of the repository/repositories and accession number(s) can be found below: https://www.ncbi.nlm.nih.gov/, TCGA-COAD, https://www.ncbi.nlm.nih.gov/, TCGA-READ, https://www.ncbi.nlm.nih.gov/, GSE17536, https://www.ncbi.nlm.nih.gov/, GSE17537, https://www.ncbi.nlm.nih.gov/, GSE29621, https://www.ncbi.nlm.nih.gov/, GSE39582, https://www.ncbi.nlm.nih.gov/, GSE38832.

## Author contributions

JCZ: Conceptualization, Data curation, Formal Analysis, Investigation, Methodology, Project administration, Resources, Software, Supervision, Visualization, Writing – original draft, Writing – review & editing. JYZ: Conceptualization, Data curation, Formal Analysis, Investigation, Methodology, Validation, Writing – review & editing. YL: Funding acquisition, Investigation, Project administration, Software, Supervision, Writing – review & editing. YZ: Conceptualization, Data curation, Project administration, Writing – review & editing. XZ: Formal Analysis, Investigation, Supervision, Writing – original draft. WC: Data curation, Formal Analysis, Visualization, Writing – review & editing. LY: Funding acquisition, Project administration, Supervision, Visualization, Writing – review & editing. QZ: Data curation, Investigation, Methodology, Resources, Supervision, Validation, Visualization, Writing – review & editing.
