# Supplementary figures and images for: Developing a machine learning-based prognosis and immunotherapeutic response signature in colorectal cancer: insights from ferroptosis, fatty acid dynamics, and the tumor microenvironment
Source: Front Immunol. 2024 Jul 15;15:1416443. doi: 10.3389/fimmu.2024.1416443 (PMC11284049; doi:10.3389/fimmu.2024.1416443)

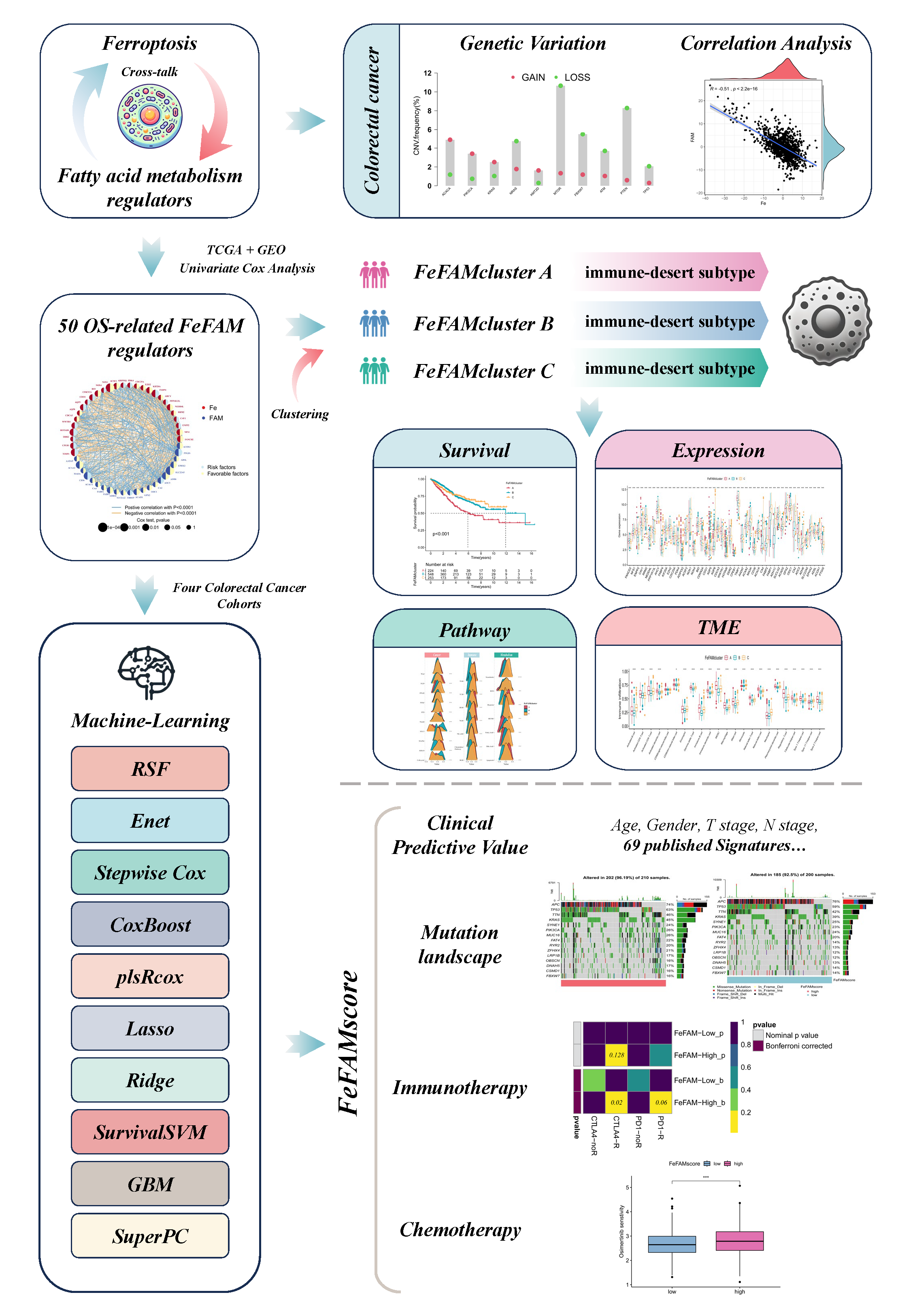

Supplement: Supplementary file 1 [file Image_1.tif]

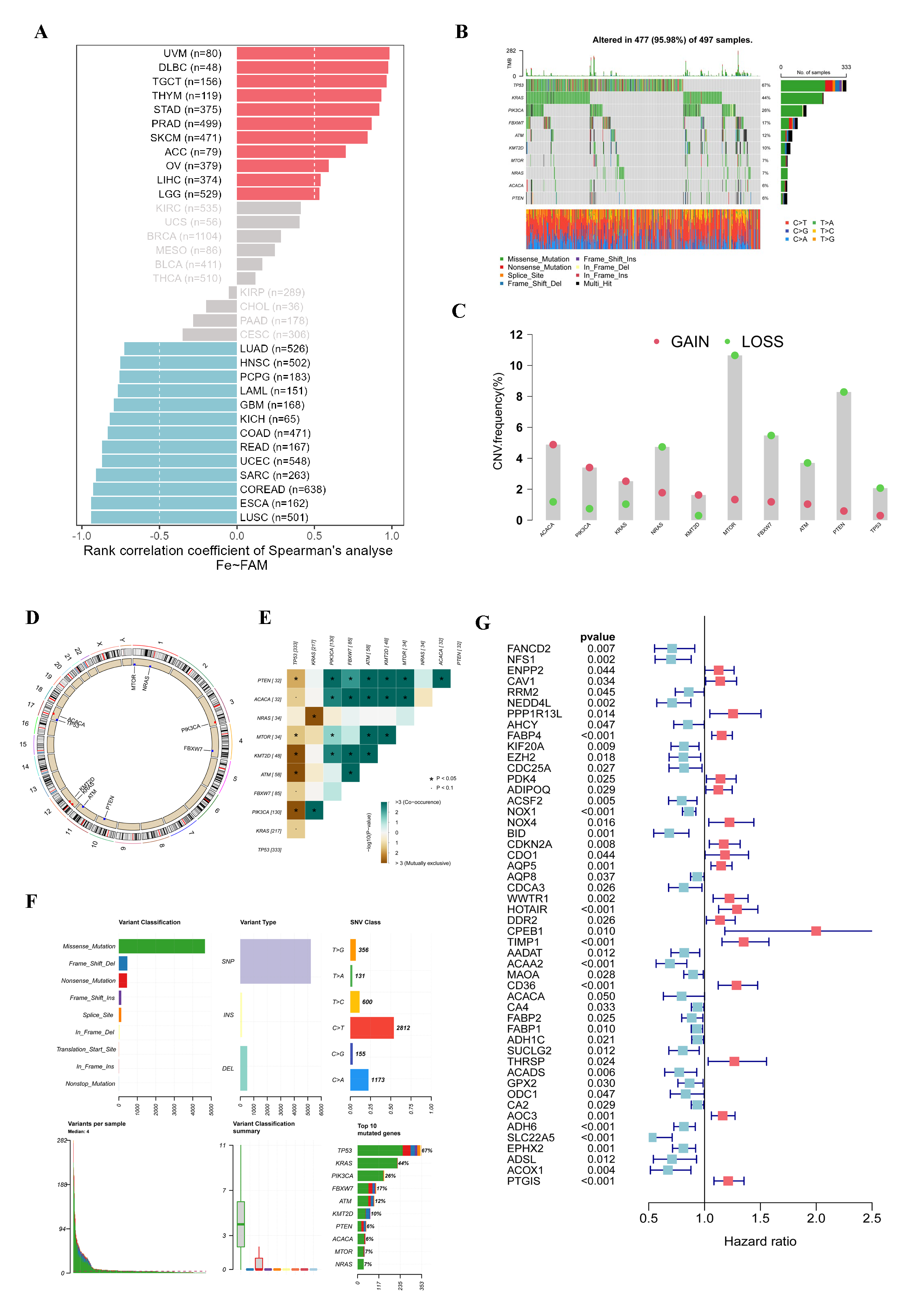

Supplement: Supplementary file 2 [file Image_2.tiff]

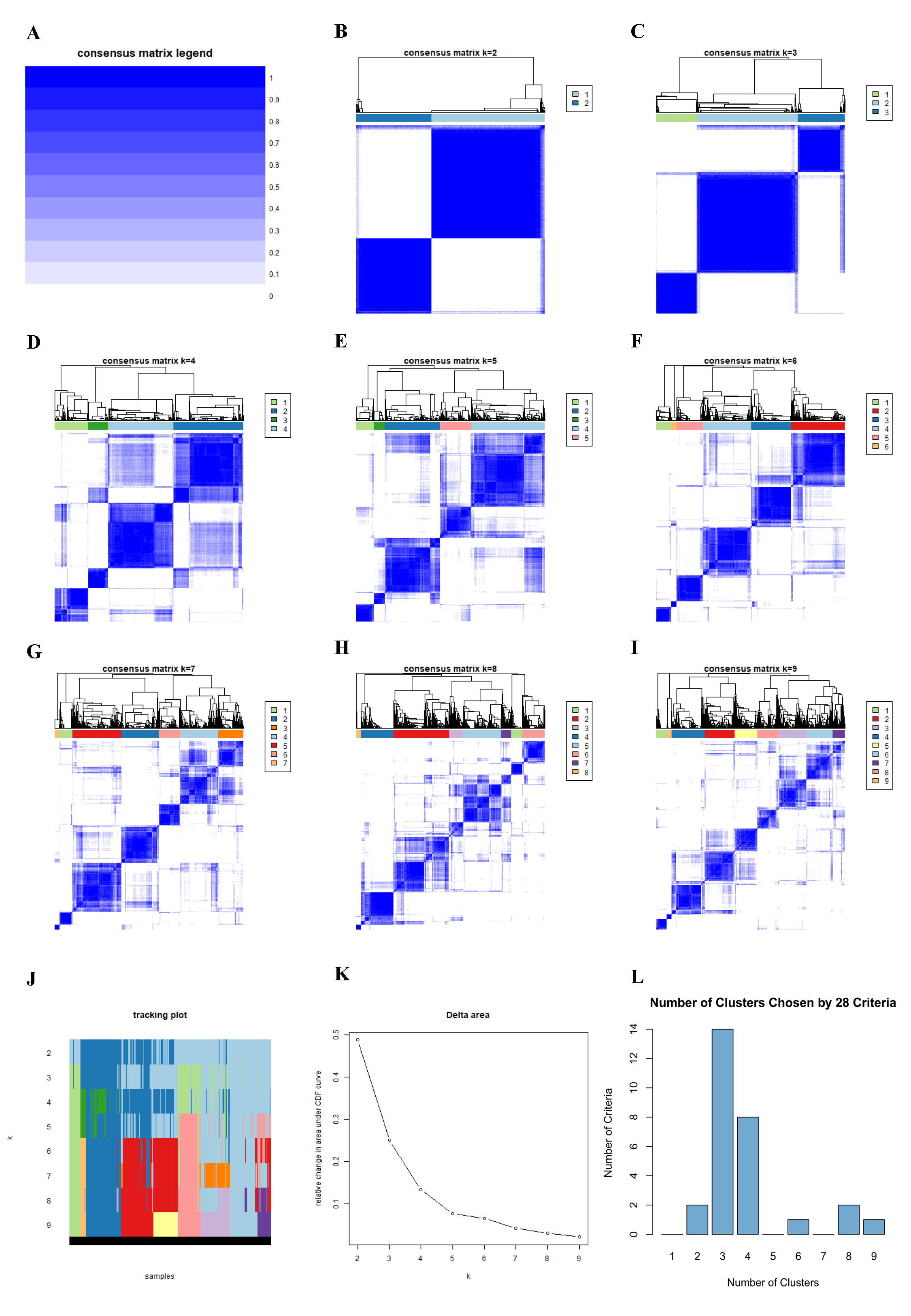

Supplement: Supplementary file 3 [file Image_3.tif]

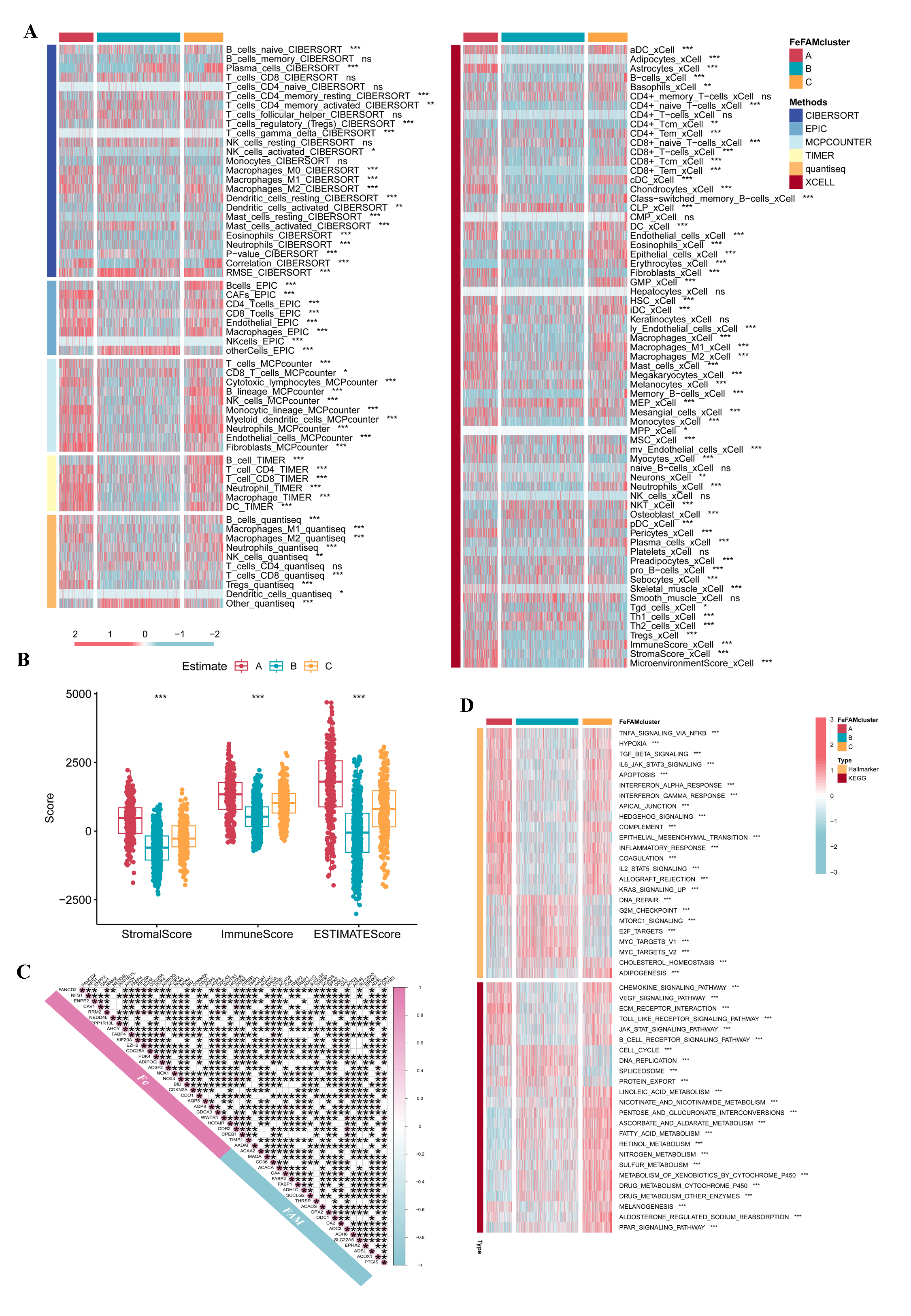

Supplement: Supplementary file 4 [file Image_4.tif]

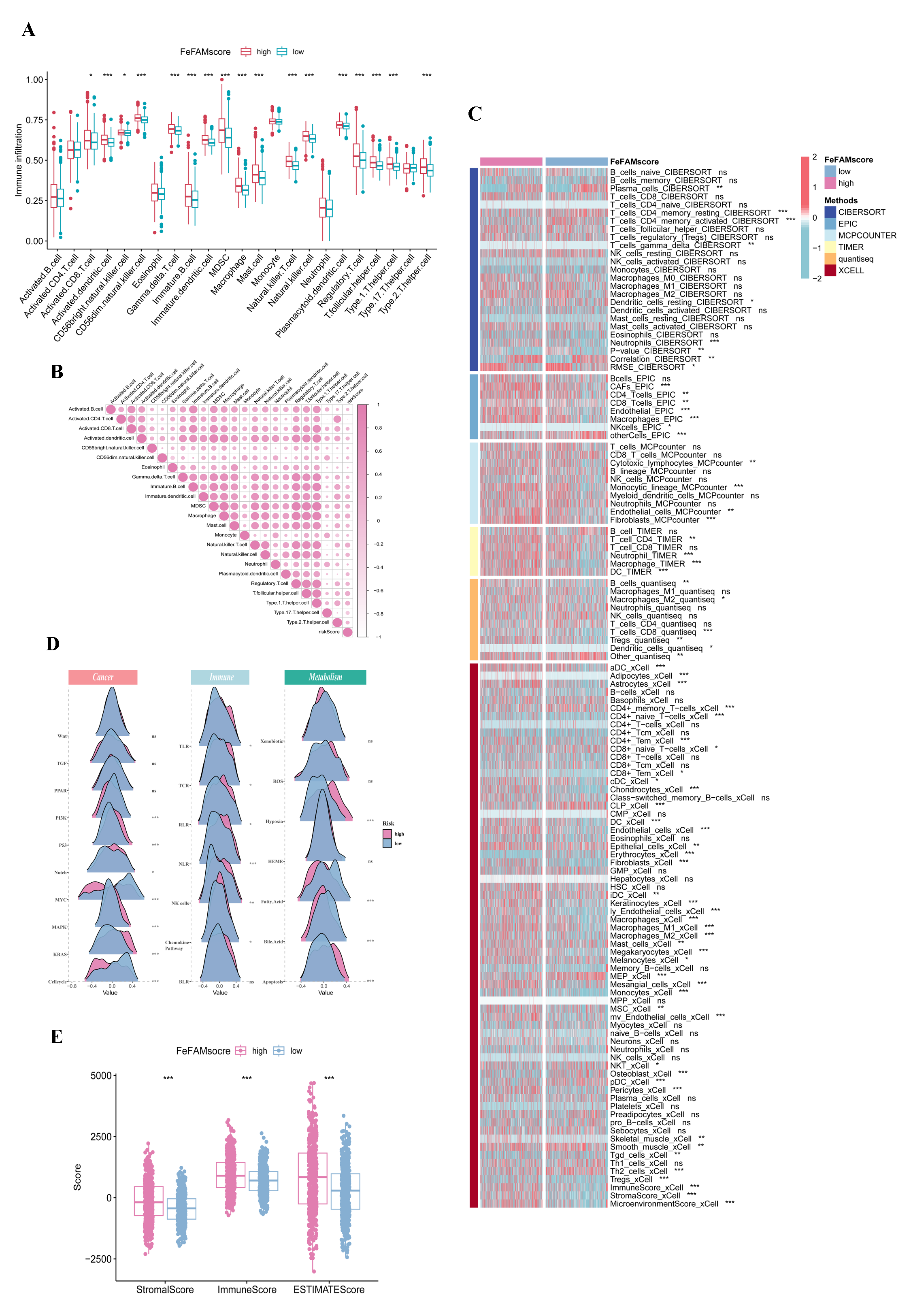

Supplement: Supplementary file 5 [file Image_5.tif]

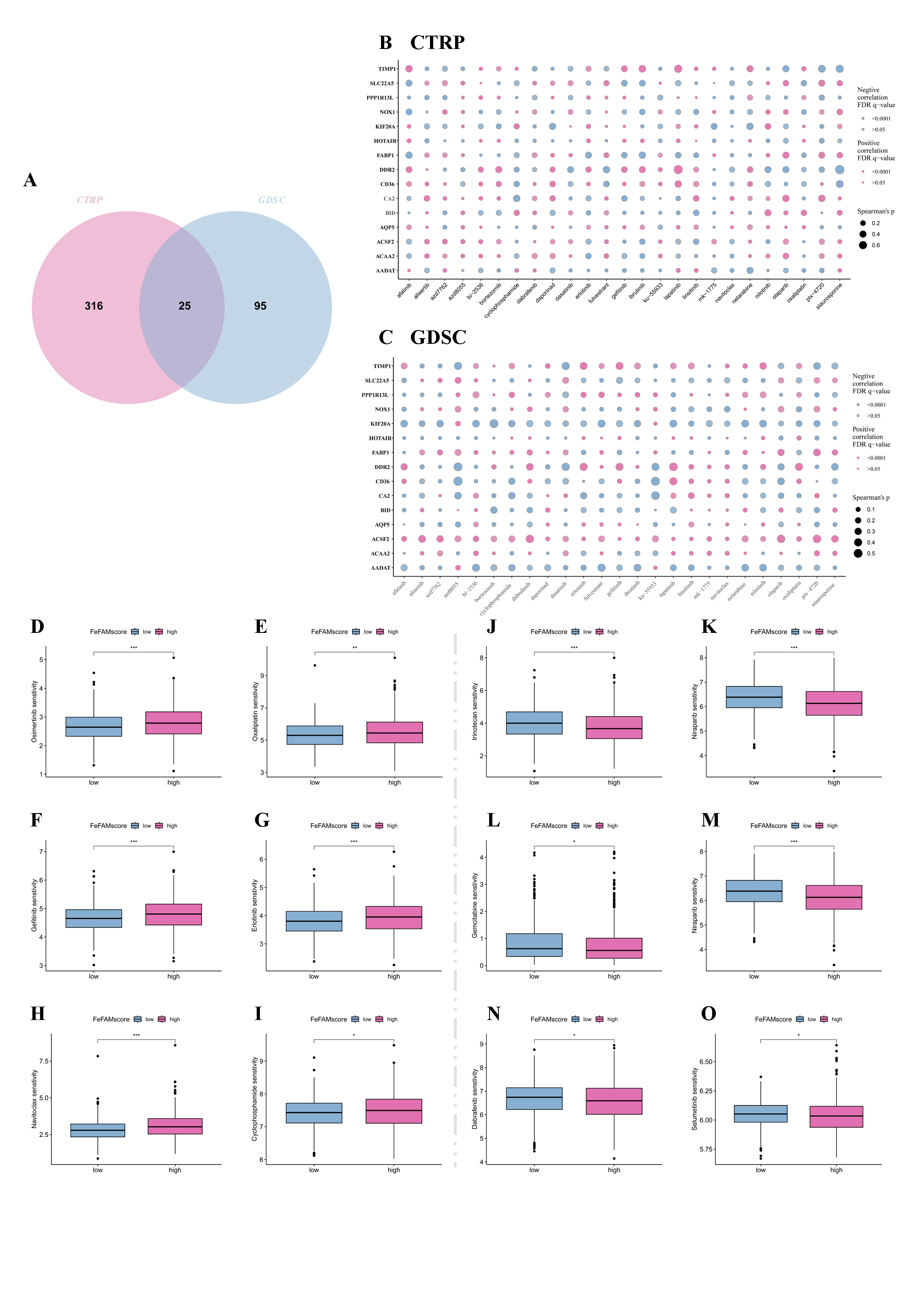

Supplement: Supplementary file 6 [file Image_6.tif]
